# Supplementary material for: Diatom identification including life cycle stages through morphological and texture descriptors
Source: PeerJ. 2019 Apr 25;7:e6770. doi: 10.7717/peerj.6770 (PMC6487182; doi:10.7717/peerj.6770)
Supplement: Supplemental Information 1 — Links to all software packages used in this paper. [file peerj-07-6770-s001.pdf]

Code availability links (11 October 2018):

Elliptical Fourier Descriptors:
https://github.com/BielStela/Elliptic-Fourier-Python/blob/master/Elliptic%20Fourier%20Descriptors.ipynb

Phase Congruency Descriptors:
https://www.peterkovesi.com/matlabfns/#phasecong

Log Gabor Filters:
http://www.iv.optica.csic.es/page49/page16/page16.html

Dimensionality Reduction:
http://scikit-learn.org/stable/auto_examples/decomposition/plot_pca_3d.html
http://scikit-learn.org/stable/modules/lda_qda.html

Clustering Algorithms and Metrics:
http://scikit-learn.org/stable/modules/clustering.html#clustering

Classificators:
http://scikit-learn.org/stable/supervised_learning.html#supervised-learning
